# Scanning electron microscopy of *Onchocerca fasciata* (Filarioidea: Onchocercidae) adults, microfilariae and eggs with notes on histopathological findings in camels

**DOI:** 10.1186/s13071-020-04123-0

**Published:** 2020-05-13

**Authors:** Younes Ghahvei, Mohammad Mirzaei, Shadi Hashemnia, Mehdi Golchin, Reza Kheirandish, Shigehiko Uni, Jairo Alfonso Mendoza-Roldan, Domenico Otranto, Alireza Sazmand

**Affiliations:** 1 Department of Pathobiology, Faculty of Veterinary Medicine, Shahid Bahonar University of Kerman, 7616914111 Kerman, Iran; 2 Department of Basic Sciences, Faculty of Veterinary Medicine, Shahid Bahonar University of Kerman, 7616914111 Kerman, Iran; 3 Department of Public Health, Faculty of Nursing, Kobe Women’s University, Kobe, 650-0046 Japan; 4 Department of Veterinary Medicine, University of Bari Aldo Moro, Str. prov. per Casamassima km 3, Valenzano, 70010 Bari, Italy; 5 Department of Pathobiology, Faculty of Veterinary Science, Bu-Ali Sina University, 6517658978 Hamedan, Iran

**Keywords:** *Onchocerca fasciata*, *Camelus dromedarius*, *Camelus bactrianus*, Vector-borne disease, Scanning electron microscopy, Histopathology

## Abstract

**Background:**

*Onchocerca fasciata* is a prevalent filarial species in camelids of Asia and Africa forming nodules in the skin of dromedary and Bactrian camels. In spite of recent advances in the biology and epidemiology of this nematode species, a relatively scant number of studies have focussed on the morphology of this parasite. The main objective of this study was to describe morphological characteristics of adults, microfilariae and eggs of *O. fasciata* by scanning electron microscopy (SEM), staining and histology.

**Methods:**

From April 2016 to March 2017 dromedary camels (*n* = 456) were inspected for infection with *O. fasciata* in a slaughterhouse in Kerman (south of Iran). Adult worms in nodules were isolated by digestion of nodules in collagenase and used for SEM. Skin nodules were also fixed, sectioned and stained with hematoxylin and eosin for histopathology. Skin microfilariae that were isolated from tissues surrounding the nodules were confirmed as *O. fasciata* by sequencing of the cytochrome *c* oxidase subunit 1 (*cox*1) and *12S* rRNA genes and used for SEM and Giemsa staining.

**Results:**

Single or multiple *O. fasciata* nodules (1.2–2.2 cm in diameter and 507–845 mg in weight) were found in 30.3% of the examined camels. SEM analysis helped identify 18 papillae in the caudal region of the male. Discontinuous longitudinal cuticular crests were observed in the posterior region of the male. In female nematodes, the ridges had a rounded shape with a height/width ratio of 7/16 in longitudinal sections. Unsheathed skin microfilariae with a rounded anterior extremity measured 210.7 × 2.5 μm on average. Developed eggs containing microfilariae measured 35.9 × 31.0 μm and their smooth shell surface had characteristic tongue-like appendages. In addition to inflammatory reactions surrounding the parasites, accumulation of intracellular ceroid pigment, golden-yellow to brown in colour, was observed within macrophages upon histopathological examination.

**Conclusions:**

We found longitudinal crests on the surface of the posterior region of the male nematode. Measurements of the main morphological features of microfilariae and eggs, and the shape index of ridges (height/width) in female nematodes are described for the first time.
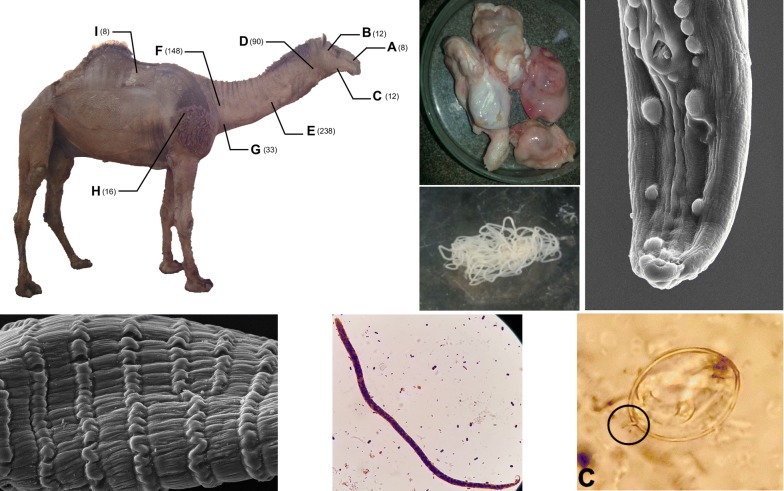

## Background

Dromedary camels are important to the economy of many countries in arid and semi-arid areas of the world. With a worldwide population of over 36 million and distribution in 47 countries they are important food sources which are productive under harsh conditions [1]. Nonetheless, there is shortage of knowledge regarding helminths that develop exclusively in camels. *Onchocerca fasciata* Railliet & Henry, 1910 is a prevalent parasite of subcutaneous connective tissue and nuchal ligament in both dromedary (one-humped; *Camelus dromedarius*) and Bactrian camels (two-humped; *Camelus bactrianus*) in Asia (e.g. China [2], Iran [3], Jordan [4], Pakistan [5], Saudi Arabia [6], Turkmenistan [7], Uzbekistan [8]) and Africa (e.g. Egypt [9], Ethiopia [10] and Sudan [11]). This species forms nodules with a self-limiting nature, but severe infections cause considerable economic losses due to condemnation of carcasses [12]. Moreover, the nodules may sometimes be mistaken for tubercle granulomas which can result in unnecessary and wasteful condemnation of carcasses [13]. Since the original description of *O. fasciata* in 1910 the information about morphology of this parasite has been scant until Bain & Nasher re-described adult males and females in 1981 [14]. In recent years, morphological and molecular characteristics of this nematode have received attention [2, 15] including studies about the identification of potential blood-sucking insect vectors in China [16]. However, there are still gaps in the knowledge about the morphological features of this filarial nematode. In this article, we describe the morphological characteristics of adult worms, microfilariae and eggs of *O. fasciata* using scanning electron microscopy (SEM), staining and histology. In addition, histopathological alternations around the nodules and monthly occurrence of nodules in camels in an endemic area are also reported.

## Methods

### Study area

In Iran there are 189,600 camels distributed in several provinces in the east, south and centre of the country [17], being mainly used for meat production with 25,000 camels slaughtered in the country in 2018, producing 5070 tons of meat [18]. Kerman Province is located on a high margin of Kavir-e Lut (Lut Desert; one of the hottest regions in the world) in the south-central part of Iran. It has a cold desert climate with long and extremely hot summers and mild winters. The average annual rainfall is 135 mm and the temperature reaches 46 °C in summer. Over 8200 camels are distributed in various regions of the province.

### Collection of specimens

#### Isolation of nodules

From April 2016 to March 2017 a total of 456 dromedary camels (258 males and 198 females) aged 1–12 years-old were slaughtered in local abattoirs and examined *post-mortem* for the presence of *O. fasciata* nodules. The number, distribution pattern and size of the nodules were recorded for each camel. Nodules were collected and transferred to the Laboratory of Parasitology, Faculty of Veterinary Medicine, Shahid Bahonar University of Kerman, Iran.

#### Isolation and staining of microfilariae

Tissues surrounding the nodules were carefully removed, placed in a centrifuge tube containing physiological saline, and incubated overnight at room temperature. Then pieces of tissue were removed and the tube was centrifuged at 1000× *rpm* for 10 min. Droplets of the sediment were microscopically examined for the presence of microfilariae of *O. fasciata* [14]. Giemsa staining was also used for description of the main morphological features of microfilariae. For this purpose, the sediment was transferred onto a slide, fixed with methanol and air-dried. The slides were placed upside-down in a 1:20 dilution of commercial Giemsa solution for 20 h and then gently rinsed in tap water to remove the excess stain. One portion of the recovered microfilariae were fixed in glutaraldehyde and osmium tetroxide for further processing for scanning electron microscopy, and another portion was transferred to − 20 °C for PCR confirmation.

#### Isolation of adult worms and preparation for scanning electron microscopy

Adult worms were isolated from nodules after digestion with type I collagenase [15]. Recovered worms were fixed with glutaraldehyde and osmium tetroxide. The specimens were then dehydrated in a graded series of ethanol and mounted on stubs. The SEM preparation was continued by sputter coating the specimens with gold (Agar Scientific Ltd., Essex, UK) in a SC7620 fine coater (Quorum Technologies, East Sussex, UK). Finally, the micrographs were prepared using a LEO1450VP scanning electron microscope (Carl Zeiss AG, Oberkochen, Germany) operating at 20 kV.

### Molecular identification of microfilariae and PCR assay

To confirm the morphological identification of the microfilariae, their genomic DNA was extracted using the High Pure PCR Template Preparation Kit (Roche, Basel, Switzerland), and PCRs targeting two mitochondrial genes: cytochrome *c* oxidase subunit 1 (*cox*1) [19] and small-subunit ribosomal DNA (*12S* rDNA) [20] were performed. PCR products were directly sequenced in both directions and the sequences were deposited in the GenBank database.

### Histopathology

Three nodules and their surrounding tissues were fixed in 10% neutral buffered formalin solution, embedded in paraffin, sectioned at a 5 μm thickness, stained with haematoxylin and eosin and studied under a light microscope.

### Statistical analysis

Chi-square tests were performed using SPSS ver. 20 (SPSS Inc., Chicago, USA) to determine statistical differences in the prevalence of *O. fasciata* between camels of different ages and sexes. *P*-values < 0.05 were considered significant.

## Results

### Prevalence of onchocercosis

*Onchocerca fasciata* nodules were observed in 138 (30.3%) out of 456 examined dromedary camels. Nodule intensity ranged between 1–9 with a mean intensity of 4.12 (95% CI: 3.8–4.5) and mean abundance of 1.24 (95% CI: 1.06–1.45). Nodules ranged between 1.2–2.2 (1.51) cm in diameter and 507–845 (604) mg in weight. Infection was not statistically different between male (72/258, 27.9%) and female (66/198, 33.3%) camels (*χ*^2^ = 1.563, *df* = 1, *P* = 0.211) or in camels over 3 years of age (108/354, 30.5%) in comparison with younger camels (30/102, 29.4%) (*χ*^2^ = 0.045, *df* = 1, *P* = 0.832). The ratio of infected camels *vs* non-infected camels was highest in September and August, and lowest in March and February although this difference was not significant (Additional file 1: Table S1, Additional file 2: Figure S1).

### Distribution of *O. fasciata* nodules

**Fig. 1:**
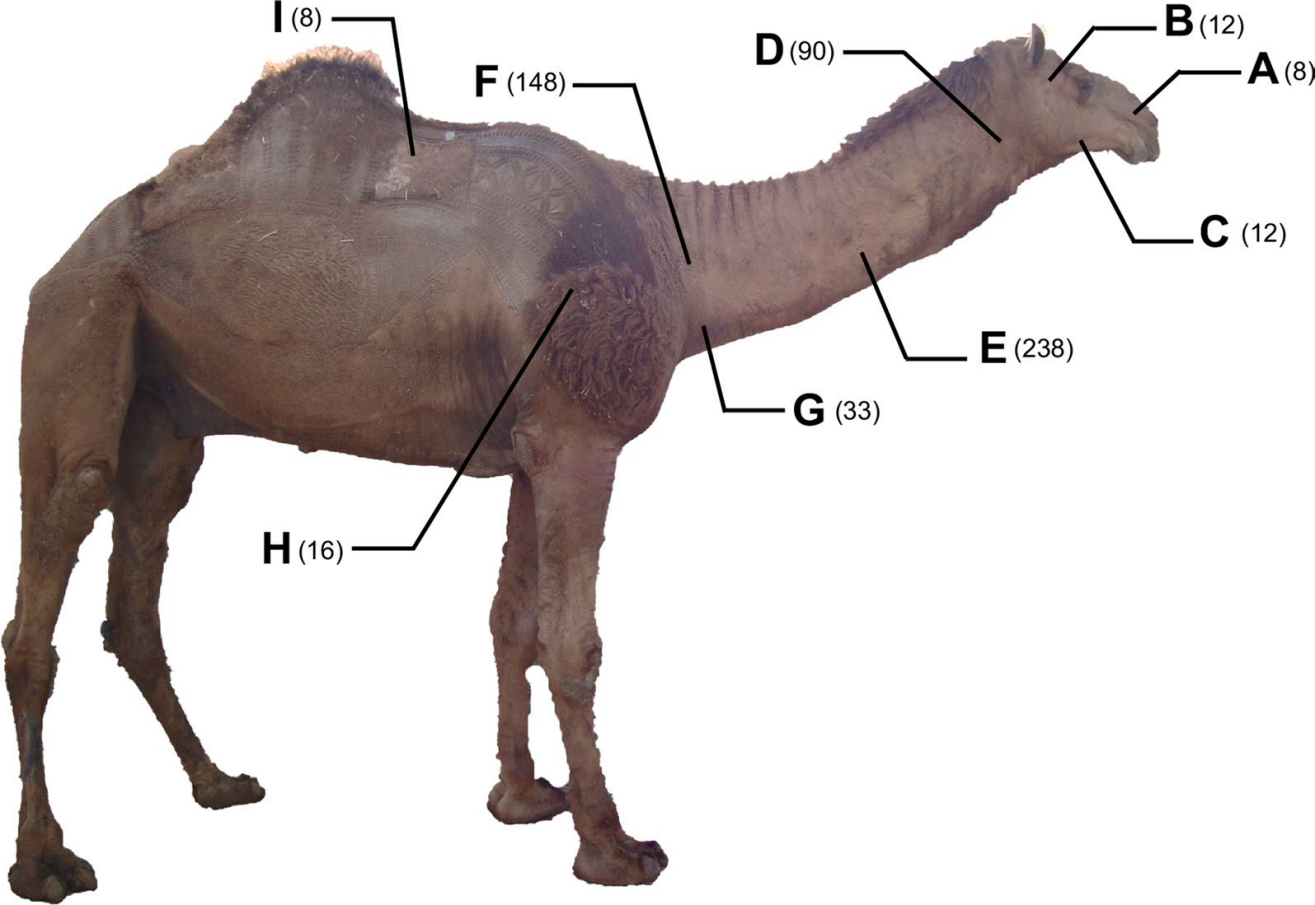
Distribution of 565 *O. fasciata* nodules on the body of 138 dromedary camels in Iran

A total of 565 skin nodules were observed. As shown in Fig. 1, nodules were more prevalent on the sides of the neck (90.1%) [areas E and F had the highest prevalence (16.2% and 12.5%, respectively) and mean intensity (3.2 and 2.6, respectively)] followed by the head (5.7%) [areas A, B and C], scapular region (2.8%) [area H] and below the hupms (1.4%) [area I]. No nodules were observed in nuchal ligament area.

### Morphology and anatomy observations

#### Male

**Fig. 2:**
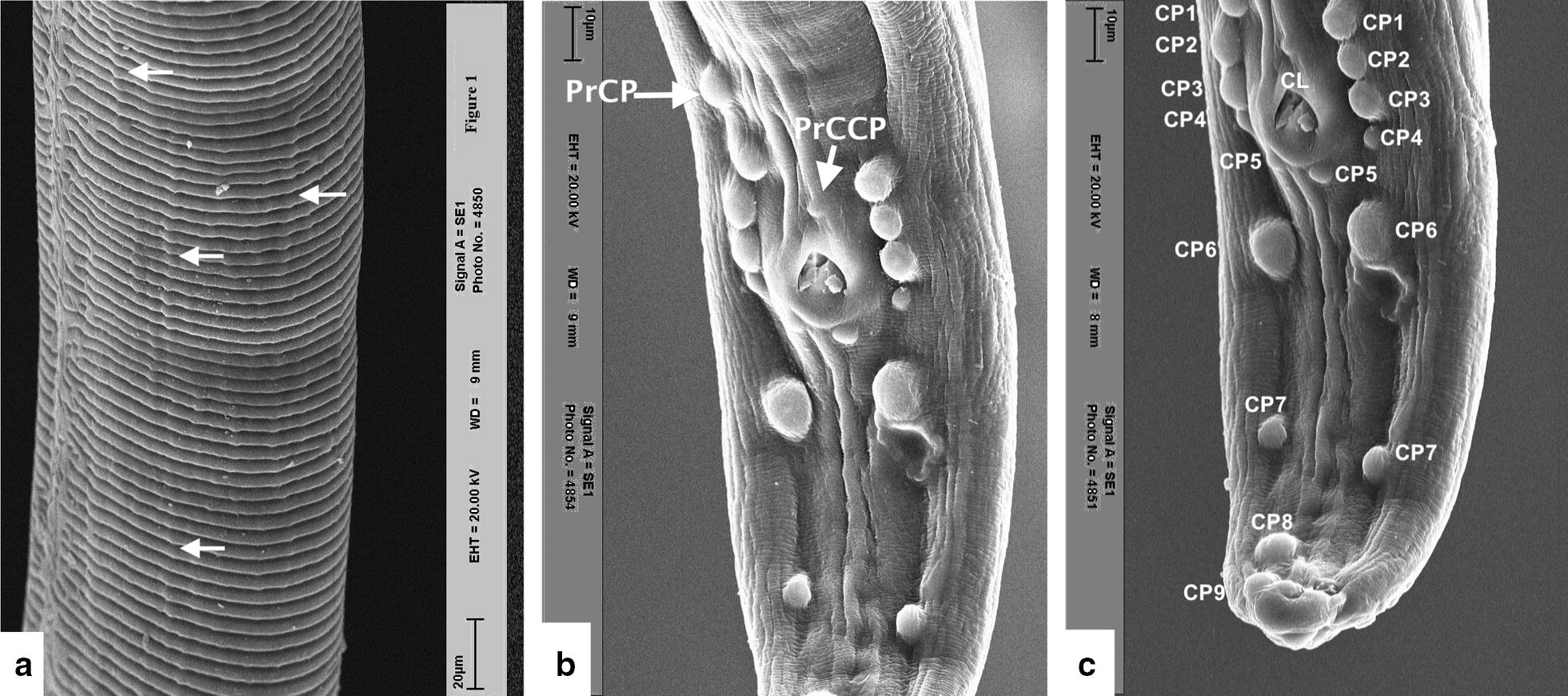
SEM images of male *O. fasciata*. **a** Longitudinal crests (arrows) in the posterior part. **b** Precloacal region with an unpaired precloacal papilla (PrCP) and a precloacal central papilla (PrCCP). **c** Posterior region with caudal papillae (CP) and cloaca (CL). *Scale-bars*: **a**, 20 µm; **b**, **c**, 10 µm

Specimens were approximately 9 cm in length and the posterior 3 cm portion was used for electron microscopy examination. Thin transverse ridges were observed on the cuticle of the midbody becoming closer and narrower towards the two extremities. The striation met at the lateral line. Some discontinuous longitudinal cuticular crests were observed in the posterior region (Fig. 2a). Posterior extremity bears 18 caudal papillae arranged as follows: two unpaired precloacal papillae (the first lateral papilla (PrCP) and the second central papilla (PrCCP) in Fig. 2b); three large precloacal pairs, CP1-3; small pairs 4 and 5 (CP 4 lateral to cloaca and CP 5 just posterior to cloaca); CP 6 larger than all others; CP 7 in the posterior third of the tail; unpaired papillae 8 and 9, close together (Fig. 2c). The two spicules were not observed in the cloacal opening.

#### Female

**Fig. 3:**
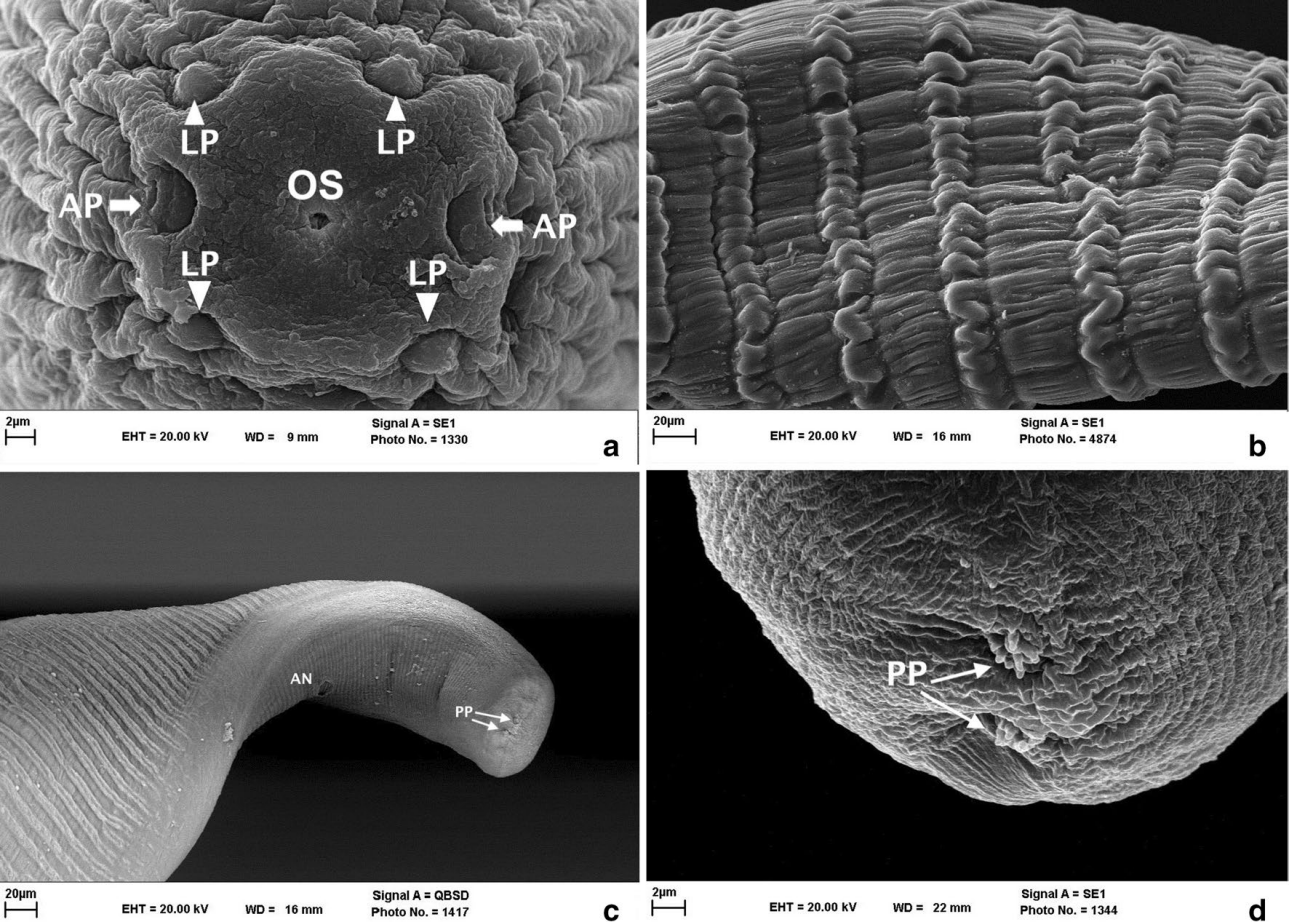
SEM images of female *O. fasciata*. **a** Apical view of the anterior region. Note the mouth opening (OS), two amphidial pores (AP) and four labial papillae (LP). **b** Transverse cuticular ridges at midbody. **c** Posterior region. Note the anal pore (AN) and phasmidial pores (PP). **d** Higher magnification of plasmids. Note the cilia exposed to the outside. *Scale-bars*: **a**, **d**, 2 µm; **b**, **c**, 20 µm

Two lateral amphidial pores and four labial papillae were observed at the anterior extremity (Fig. 3a). The outer layer of the cuticle comprised thick transverse ridges at midbody, which became narrower and closer towards the two extremities as in the males. Ridges appeared as rough undulating lines, which were interrupted in the lateral sides of the body, and bifurcated in some cases (Fig. 3b). The pointed and spirally-coiled posterior extremity of a female *O. fasciata* is shown in Fig. 3c and the phasmidial pores in Fig. 3d. Histological examination showed a distance between 2 adjacent ridges of 84.4–106.9 (mean 90.8) μm; the shape of the ridges was rounded with a height/width ratio of 7/16 in longitudinal sections; and the distance between 2 adjacent striae was 33.9 μm. For comparison of the shape of the ridges, a previously published photomicrograph from the midbody region of a female *O. fasciata* was assessed (figure 1D in [15]). Although the shape of the ridges was rounded, a height/width ratio of 10.1/20.1 was recorded from this published figure.

#### Microfilaria

**Fig. 4:**
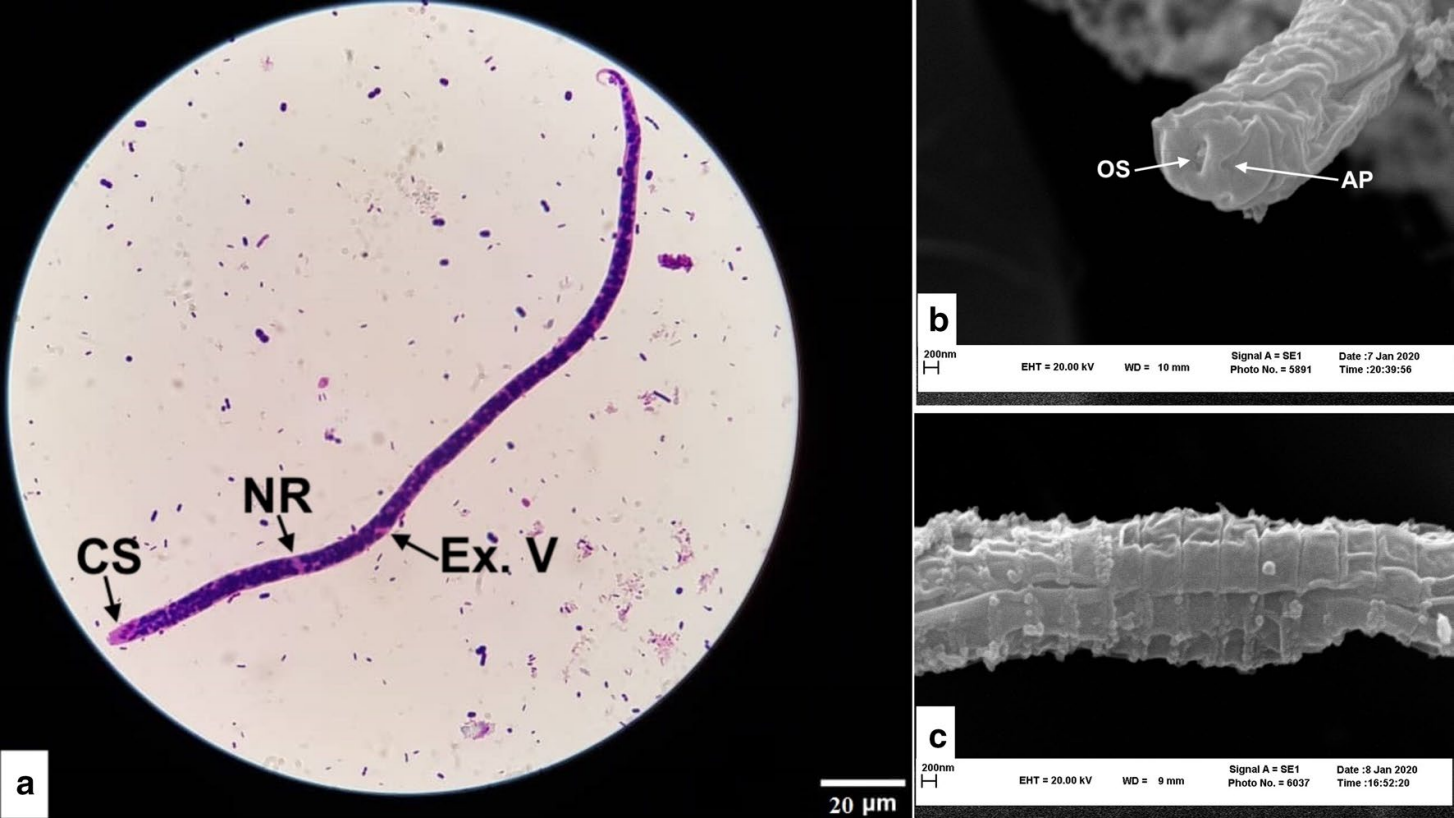
Morphological features of *O. fasciata* microfilaria. **a** Giemsa staining of a microfilaria isolated from tissue surrounding the skin nodule. Note the short cephalic space (SC), nerve-ring (NR) and excretory vesicle (Ex. V). **b** SEM micrograph of the anterior region. Note the mouth opening (OS) and amphid (AP). **c** SEM micrograph of the midbody region showing conspicuous striations. *Scale-bars*: **a**, 20 µm; **b**, **c**, 200 nm

Unsheathed microfilariae were recovered enclosed in the *Onchocerca* nodules. Body length of 20 analysed microfilariae ranged between 197.5–239.6 (mean 210.7) μm, and body width ranged between 1.8–3.6 (mean 2.5) μm. Microfilariae had a rounded anterior extremity with a cephalic space free from nuclei and measuring 3.1–3.5 (mean 3.3) μm. The nerve-ring was located at 45.8–57.4 (mean 51.6) μm from the anterior extremity. No nuclei were observed at the fine-pointed tail; the last posterior nucleus was located at 6.4–8.4 (7.4) μm from the tip of the tail. The column of body nuclei was compact (Fig. 4a). In SEM micrographs, rounded anterior extremity (Fig. 4b), conspicuous striations (Fig. 4c) and a fine tip were observed.

#### Eggs

In one preparation, eggs containing microfilariae were recovered which was probably due to rupturing of a female nematode. The shape of the eggs was rounded and the surface was smooth (Additional file 3: Figure S2a). The microfilariae were located inside the eggs with different spaces from the shell surface according to development stage and measured on average 35.9 × 31.0 μm (*n* = 20 developed eggs) (Additional file 3: Figure S2b). Egg-shell had characteristic tongue-like appendages (Additional file 3: Figure S2c).

### Pathology

A variety of histopathological lesions induced by adult forms of *O. fasciata* were observed from the examined specimens. General lesions such as haemorrhage and congestion were visible. There were several cavities containing transverse sections of adult nematodes. Some fragments of the worms were quite recognizable including the cuticle, musculature, the intestine, and the uterus, sometimes with embryonated eggs inside. These female nematodes were surrounded with heavy infiltration of polymorphonuclear neutrophils, eosinophils and necrotic debris followed by lymphocytes, plasma cells, macrophages, epithelioid cells, multinucleated giant cells and connective tissue. With time, the adult parasites in the centre of the cavities with surrounding inflammatory cells underwent coagulative necrosis due to the deleterious effects of the parasite. Dystrophic mineralization and calcification occurred following the necrosis of the parasites (Additional file 4: Figure S3). As a result of tissue damage and necrosis, ceroid golden-yellow to brown intracellular pigment accumulated within the macrophages recruited in the damaged areas, also with fibrosis. No direct lesion attributed to *O. fasciata* microfilariae was found in the skin of the infected camels.

### PCR and sequencing

Nucleotide sequences of *12S* rRNA and *cox*1 genes of the microfilariae that were used for SEM confirmed their identification as *O. fasciata*. The sequences were deposited in the GenBank database under the accession numbers: MT002817 (*cox*1) and MN985127-MN985128 (*12S* rDNA). BLASTn results for both gene loci revealed that *O. fasciata* in this region had 99% similarity with isolated nematodes from dromedary camels in Iran (GenBank: MG188678-MG188679) and Saudi Arabia (GenBank: DQ523744), and 100% identity with a nematode from a Bactrian camel in China (GenBank: JQ316672).

## Discussion

In this study, SEM, histology and Giemsa staining were used to describe adult male and female, and microfilariae of *O. fasciata*. Pathological lesions related to infection of camels with *O. fasciata* were also described. The rate of infection of examined dromedary camels with onchocercal nodules was 30.3% which is within the range recorded in previous studies on dromedary camels in Iran (5.8–48.0%; [21]). Infection of dromedaries is reported to be 26.2–59.0% in Saudi Arabia [6, 22, 23], 2.0–23.1% in Jordan [4, 12, 24] and 2.7% in Egypt [9]. Also, 49.0–80.3% of Bactrian camels in China have been diagnosed with *Onchocerca* nodules [2, 16]. Although *O. gutturosa* and *O. armillata* which infect camels in Sudan [25, 26] and Australia [27] have been reported from cattle in Iran [28], so far there are no reports of camel onchocercosis due to these species in the country [21]. In this study, no statistical difference was observed in the infection in male and female camels; however, older camels were infected more frequently which could be due to a longer exposure to the vector. In contrast, no increase in parasite density with age was recorded in *O. gutturosa* infection in cattle, which is attributed to the immune reaction of the host species to *Onchocerca* infection [29].

With regard to monthly incidence of the infection in this endemic area in the eastern Mediterranean region, onchocercosis peaked in August and September. A monthly peak of *O. fasciata* infection was recorded in September in Saudi Arabia [22] and July to August in Egypt [9]. Currently there is no information available on: (i) the time required for ingested microfilariae to develop to the infective stage in the arthropod vector; (ii) the pre-patent period of infection; and (iii) the development of adults in the nodules. Further research on these three major issues is required.

Similar to the present finding on the distribution of nodules on the body of camels, all studies from Iran have reported that the side of the neck is the most affected area [3, 30, 31]. In contrast, in Saudi Arabia and China, the nuchal ligament is the area where the majority of nodules are detected [2, 23], but in our study no nodules were observed in the nuchal ligament area. As a close relationship between the biting habits of the vectors and the distribution of onchocercal nodules have been demonstrated [32], the difference in location of nodules in camels of various localities might be related to dipteran vectors in these regions. The vector of *O. fasciata* has been identified in Inner Mongolia, China, where out of 49 aquatic dipteran insects in the living environment of Bactrian camels, only *Culicoides puncticollis* was found infected with larvae of this nematode [16]. It is known that, generally, species of the genus *Onchocerca* are transmitted either by simuliids or by ceratopogonids [33]. For instance in a study where both *Simulium ornatum* and *Culicoides nubeculosus* were experimentally infected with L1 of *Onchocerca lienalis* and *O. gutturosa*, development of *O. lienalis* to L3 occurred only in *S. ornatum*, whereas *O. gutturosa* L3 developed only in *Culicoides nubeculosus* [34]. Regarding *O. fasciata*, finding of a *Culicoides* species infected with larvae [16], and the fact that camels usually live in arid and semi-arid areas with no flowing water (suitable for *Simulium* spp. breeding) gives weight to the hypothesis that *O. fasciata* is transmitted by midges and not by blackflies.

The SEM observation of the male caudal region revealed an arrangement of 18 papillae which differs from previous descriptions. In their comprehensive redescription in 1981 Bain & Nasher [14] mentioned 18 or 19 papillae with no medioventral papillae posterior to the cloaca. Recently, in Bactrian camels of China, male *O. fasciata* was described with eight to nine pairs of caudal papillae arranged as two large precloacal pairs, two subventral small pairs, one lateral large postcloacal pair and three aligned pairs in the posterior tail region [2]. The most instructive character in regard to morphological and biological evolution of *Onchocerca* species is the number and arrangement of the caudal papillae [35]. Although the arrangement of caudal papillae is important for species identification, minor difference such as the location and size of the caudal papillae might be observed between specimens [36]. *Onchocerca raillieti*, the most primitive species infecting donkeys in Africa, has a perfect spirurid plan with ten pairs of caudal papillae (four precloacal, two postcloacal and four which constitute a subterminal group). In other *Onchocerca* species, a regression of the precloacal papillae, their grouping close to the cloaca, the transformation of some subterminal papillae into cuticular points, their reduction in number and migration of terminal papillae towards the cloaca has been observed [35]. Current knowledge based on morphological characters and molecular analyses suggests that *O. fasciata* is fairly primitive and did not diverge from an older independent speciation event [15, 37].

Interestingly, some discontinuous longitudinal cuticular crests were observed in the posterior region of *O. fasciata* males. Finding male *O. fasciata* is accidental as they roam in the subcutaneous tissue and usually do not participate in the formation of nodules [23, 38, 39]. In this study it was not possible to take photomicrographs from the midbody of males to find out more about these cuticular crests. Longitudinal cuticular crests are characteristic for male *O. dewittei dewittei* and *O. d. japonica* in Asian suids [40]. However, such crests were not found by SEM in the midbody of male *O. eberhardi* from sika deer in Japan [41]. Therefore, it is necessary to elucidate the taxonomic significance of the crests in the male similar to what is recognized for transverse ridges of the female.

SEM observations of female *O. fasciata* revealed that the outer layer of the cuticle comprised thick transverse ridges at midbody. Ridges appeared as rough undulating lines, which were interrupted along the lateral sides of the body, and bifurcated in some cases. Complexity of the female cuticle, which enables the worm to resist pressure by surrounding tissues such as tendons and ligaments, is a distinctive feature in species of the genus *Onchocerca*. In females of *O. fasciata*, the longest *Onchocerca* species reaching 117 cm, the cuticle differentiates into an internal layer which is divided transversely by striae and an external layer with transverse rings, called ridges [15]. Protruding and wavy cuticular ridges and their bifurcation in *O. fasciata* females observed here are consistent with previous descriptions [14, 23]. Longitudinal histological sections of a female *O. fasciata* found in a nodule excised from the neck region also showed round-shaped ridges with 2–4 striae between adjacent transverse ridges. In a previous study, two striae were observed between adjacent transverse ridges [15]. The distance between two adjacent ridges in the histosections was 90.8 µm and distance between two adjacent striae was 41.8 µm. While data available in the literature were recorded at the midbody (43 and 21 µm, respectively) of female nematodes [15], in *O. fasciata* isolated from Bactrian camels, ridges were separated by spaces of up to 69 μm [2]. In the present study, striae were 0.3–19.0 μm wide depending on the anatomical location but in our histological sections the distance between two adjacent striae ranged between 18.1–43.6 μm. This difference in morphological characters of *O. fasciata* in dromedaries and Bactrian camels, two different host species although in the same genus, needs further examinations.

Regarding the reactions to *O. fasciata* observed by histology, multiple granulomas with infiltration of neutrophils, eosinophils, lymphocytes, plasma cells, macrophage, multinucleated giant cells and fibrous connective walls surrounding the transverse and longitudinal sections of the parasite were observed in recent lesions. However, different degrees of caseous necrosis and tissue debris with degenerated or dead calcified parasites were detected in the cavities present in the older lesions. These findings are consistent with those obtained in previous studies [3, 30, 31]. However, accumulation of a golden-brown intracellular pigment, ceroid, was found within the macrophages in the present study, probably due to tissue damage and necrosis. Some filarial species of the genus *Onchocerca*, such as *O. fascita*, harbour bacterial endosymbionts *Wolbachia* spp. [39]. It has been suggested that the production of chemokines with chemotactic activity for neutrophils can be induced by *Wolbachia*-positive *Onchocerca* [42, 43]. On the other hand, there is a shift from neutrophilic to eosinophilic infiltration of inflammatory cells around the worms. However, it is not completely known whether eosinophil degranulation is initiated by the living nematode or a sustained infiltration of eosinophils kills the worms [42]. Also, it has been suggested that eosinophils might accumulate around the worms to kill them after elimination of *Wolbachia* by antiparasitic agents [43]. Recently, the name “*Candidatus* Wolbachia onchocercicola” was proposed for the *Wolbachia* endosymbionts that live in the subcutaneous and connective tissues of *Onchocerca ochengi*, *O. lupi* and *O. volvulus* [44]. It will be valuable to look into the distribution of *Wolbachia* endosymbionts in tissues of *O. fasciata* and evaluate which antibiotic (e.g. oxytetracycline and doxycycline) is more efficient in depletion of the endosymbiont and consequently death of adult nematodes and microfilariae.

In this study nucleotides sequences of *12S* rRNA and *cox*1 genes of the microfilariae revealed a high similarity with *O. fasciata* from Bactrian camels in China (GenBank: JQ316672). Based on the hypothesis of *Onchocerca* species evolution, Africa has been suggested as the origin of *O. fasciata* [37]; however, divergence and domestication of Bactrian camels took place earlier in history than one-humped dromedaries [45]. Hence Bactrian camels could have acquired the infection from dromedaries through the historical network of trade caravan routes (e.g. the Silk Road) and settled the infection in China upon their return. So far, there have been only two multilocus sequence typing studies performed on *O. fasciata* from Iran and Saudi Arabia [15, 46], thus further molecular phylogenetic analysis of *O. fasciata* from camels of different regions might shed light on the infection dynamics throughout the history.

## Conclusions

In this study, the adult male and female, microfilariae and eggs of *O. fasciata* were described by SEM, histology and Giemsa staining. Observations such as longitudinal crests in the male nematode, measurements and anatomical landmarks of microfilariae and shape index of ridges (height/width) in female nematodes are, to the best of our knowledge, reported for the first time. These findings add to our current knowledge on camels’ onchocercosis. Future studies on insect vector(s) of *O. fasciata* in Iran and planning strategies for controlling the infection by identification of selective treatment of camels are necessary.

## Supplementary information

**Additional file 1: Table S1.** Prevalence of *Onchocerca* nodules in camels of Kerman, Iran broken down by month, age groups and sex.

**Additional file 2: Figure S1.** Prevalence of *Onchocerca fasciata* nodules in camels of Kerman, categorized by month.

**Additional file 3: Figure S2.** Morphological features of *O. fasciata* egg. **a** SEM picture of one rounded egg (**e**) with a smooth surface besides a microfilaria (MF). **b** Giemsa staining of an egg, showing tongue-like structure on the egg-shell (**c**). *Scale-bars*: **a**, 2 µm; **b**, 20 µm.

**Additional file 4: Figure S3. a** Transverse section of adult female nematodes embedded into the cavities lined by inflammatory cells which (**b**) was selected (box). The parasites were observed encased in chronic inflammatory reactions (IR) with infiltrations of different inflammatory cells and connective tissue. H&E staining. **b** Cross-section of female *Onchocera faciata*. Note the presence of thin cuticle (yellow arrows) overlying the thick coelomyarian musculature (black arrow), and the intestine (blue arrow). A uterine tube (green arrow) containing eggs (arrowhead) is also visible. H&E staining. **c** Higher magnification of the transverse section of an adult female parasite (P) lined by neutrophils (stars), necrotic debris, mononuclear inflammatory cells, multinucleated giant cells (arrows), and collagen fibers. H&E staining. **d** Transverse section of a calcified dead adult nematode (CP) surrounded by inflammatory cells that underwent coagulative necrosis, and connective tissue. H&E staining. *Scale-bars*: **a**–**d**, 100 µm. *Abbreviations*: IR, inflammatory reaction; P, parasite; CP, calcified parasite.

## Data Availability

All data generated or analysed during this study are included in this published article and its additional files.

## Abbreviations

SEM: scanning electron microscopy
*cox*1: cytochrome *c* oxidase subunit 1
rRNA: ribosomal ribonucleic acid
PCR: polymerase chain reaction
CI: confidence interval
df: degrees of freedom
H&E: hematoxylin and eosin
