# Supplementary material for: Scanning electron microscopy of Onchocerca fasciata (Filarioidea: Onchocercidae) adults, microfilariae and eggs with notes on histopathological findings in camels
Source: Parasit Vectors. 2020 May 13;13:249. doi: 10.1186/s13071-020-04123-0 (PMC7218593; doi:10.1186/s13071-020-04123-0)
Supplement: Supplementary file 1 — Additional file 1: Table S1. Prevalence of Onchocerca nodules in camels of Kerman, Iran broken down by month, age groups and sex. [file 13071_2020_4123_MOESM1_ESM.docx]

**Additional file 2: Table S1.** Prevalence of *Onchocerca* nodules in camels of Kerman, Iran as categorized by month, age groups and sex.

| **Month** | **Total number of camels**  ***n* infected / *n* examined camels (%)** | **Age groups**  ***n* infected / *n* examined camels** | | **Sex groups**  ***n* infected / *n* examined camels** | |
| --- | --- | --- | --- | --- | --- |
|  |  | **Below 3 years old** | **Over 3 years old** | **Male** | **Female** |
| January | 9 / 38 (23,68) | 2 / 8 | 7 / 30 | 5/ 21 | 4 /17 |
| February | 8 / 40 (20) | 2 / 9 | 6 / 31 | 4 / 23 | 4 /17 |
| March | 7 / 43 (16,28) | 2 / 10 | 5 / 33 | 3 / 24 | 4 / 19 |
| April | 10 / 40 (25) | 2 / 9 | 8 / 31 | 5 / 23 | 5/ 17 |
| May | 13 / 35 (37,14) | 3 / 8 | 10 / 27 | 7 / 20 | 6 / 15 |
| June | 10 / 41 (24,39) | 2 / 9 | 8 / 32 | 5 / 23 | 5 / 18 |
| July | 13 / 35 (37,14) | 3 / 8 | 10 / 27 | 7 / 20 | 6 / 15 |
| August | 17 / 37 (45,95) | 3 / 8 | 14 / 29 | 9 / 21 | 8 / 16 |
| September | 19 / 39 (48,72) | 4 / 9 | 15 / 30 | 10 / 22 | 9 / 17 |
| October | 13 / 36 (36,11) | 3 / 8 | 10 / 28 | 7 / 20 | 6 / 16 |
| November | 11 / 42 (26,19) | 2 / 9 | 9 / 33 | 6 / 24 | 5 / 18 |
| December | 8 / 30 (26,67) | 2 / 7 | 6 / 23 | 4 / 17 | 4 / 13 |
| **Total** |  | **30 / 102 (29.4%)** | **108 / 354 (30.5%)** | **72 / 258 (27.9%)** | **66 / 198 (33.3%)** |
| ***p*-value** |  | *P* = 0.832 | | *P* = 0.211 | |
