# Supplementary material for: Scanning electron microscopy of Onchocerca fasciata (Filarioidea: Onchocercidae) adults, microfilariae and eggs with notes on histopathological findings in camels
Source: Parasit Vectors. 2020 May 13;13:249. doi: 10.1186/s13071-020-04123-0 (PMC7218593; doi:10.1186/s13071-020-04123-0)
Supplement: Supplementary file 2 — Additional file 2: Figure S1. Prevalence of Onchocerca fasciata nodules in camels of Kerman, categorized by month. [file 13071_2020_4123_MOESM2_ESM.docx]

**Additional file 1: Figure S1.** Prevalence of *Onchocerca fasciata* nodules in camels of Kerman, as categorized by month.
