# Supplementary figures and images for: Scanning electron microscopy of Onchocerca fasciata (Filarioidea: Onchocercidae) adults, microfilariae and eggs with notes on histopathological findings in camels
Source: Parasit Vectors. 2020 May 13;13:249. doi: 10.1186/s13071-020-04123-0 (PMC7218593; doi:10.1186/s13071-020-04123-0)

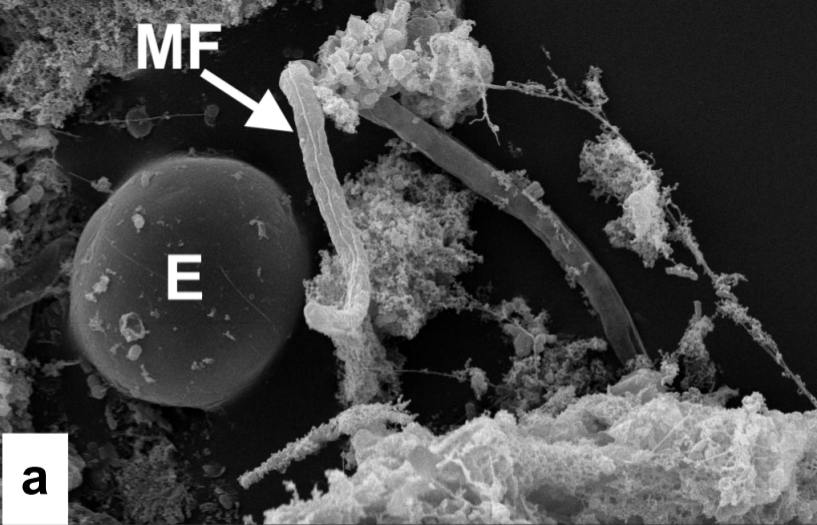

**a**

2 $\mu$ m

EHT = 20.00 kV WD = 15 mm Signal A = SE1 Date : 7 Jan 2020  
Photo No. = 5884 Time : 20:19:19

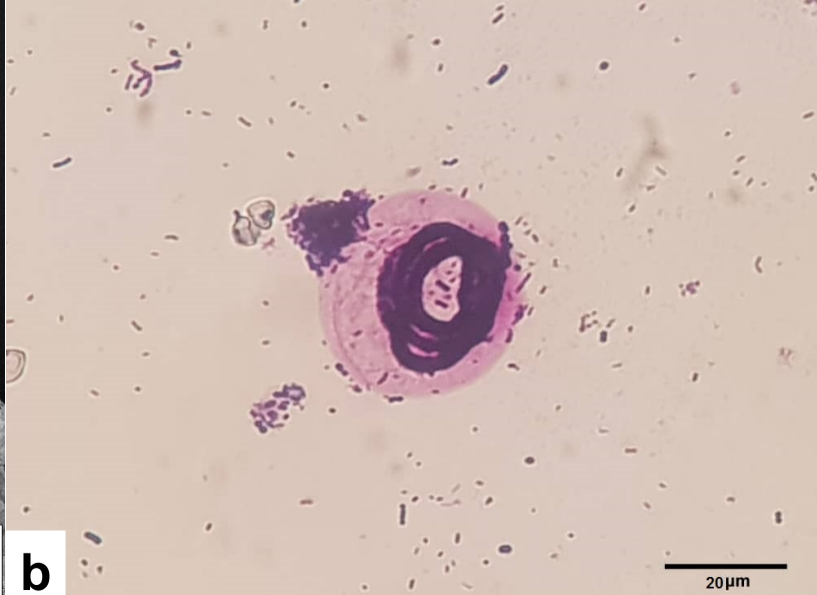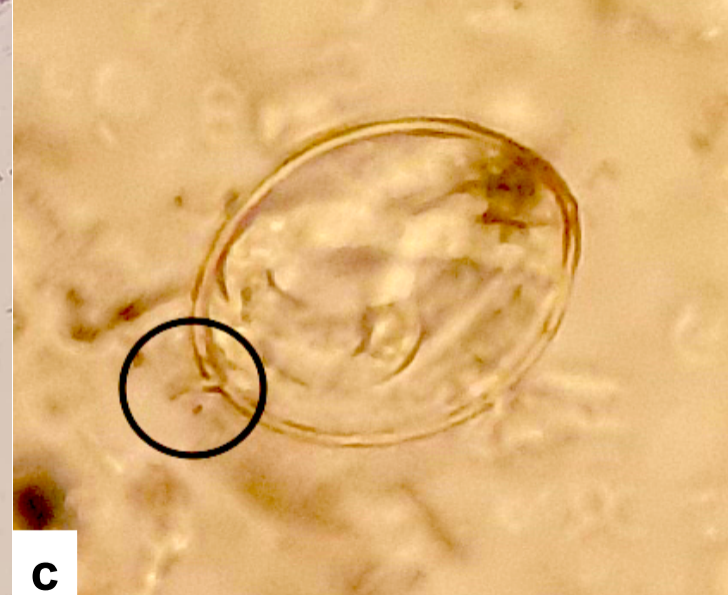

Supplement: Supplementary file 3 — Additional file 3: Figure S2. Morphological features of O. fasciata egg. a SEM picture of one rounded egg (e) with a smooth surface besides a microfilaria (MF). b Giemsa staining of an egg, showing tongue-like structure on the egg-shell (c). Scale-bars: a, 2 µm; b, 20 µm. [file 13071_2020_4123_MOESM3_ESM.pdf]

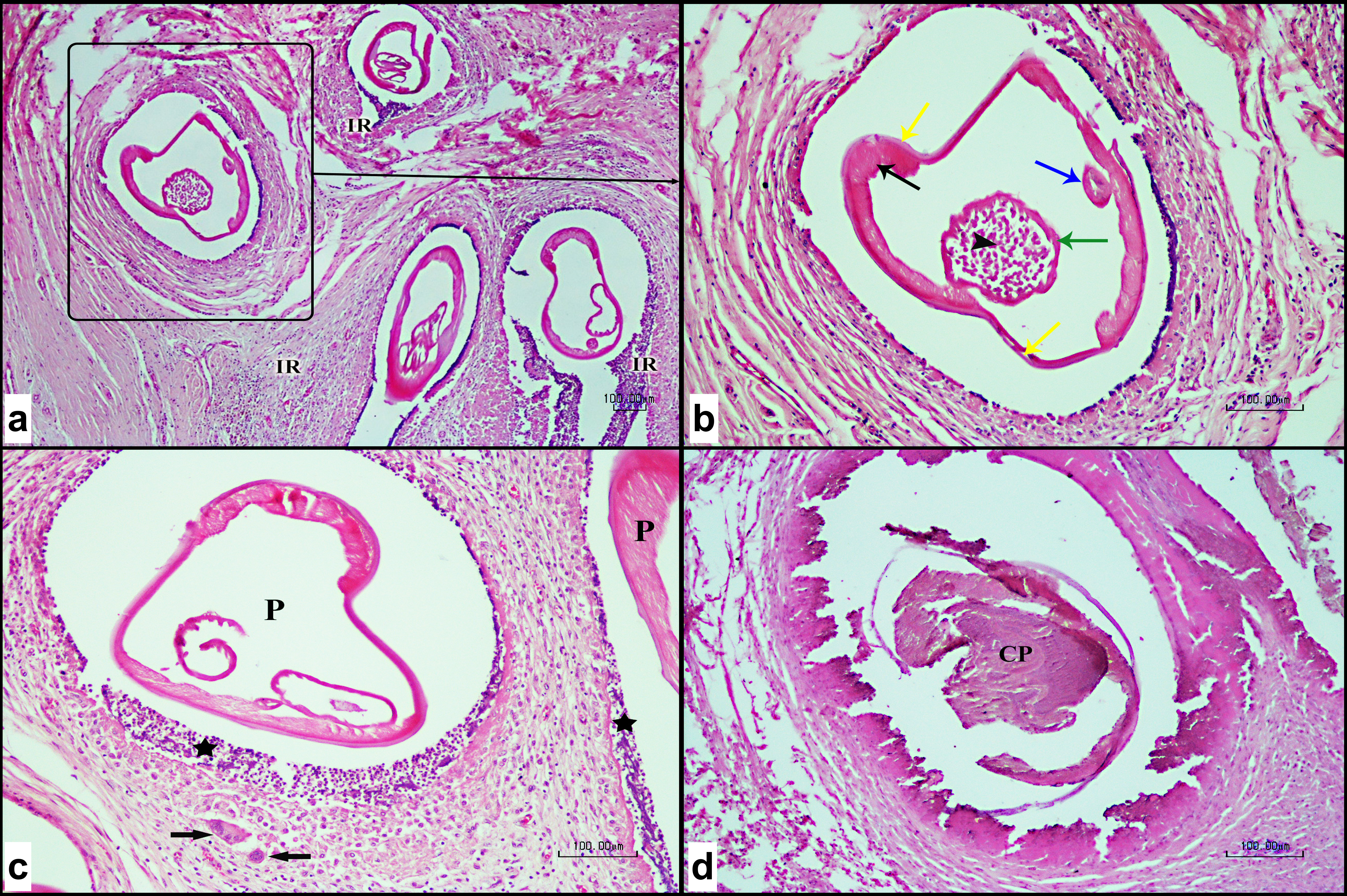

Supplement: Supplementary file 4 — Additional file 4: Figure S3. a Transverse section of adult female nematodes embedded into the cavities lined by inflammatory cells which (b) was selected (box). The parasites were observed encased in chronic inflammatory reactions (IR) with infiltrations of different inflammatory cells and connective tissue. H&E staining. b Cross-section of female Onchocera faciata. Note the presence of thin cuticle (yellow arrows) overlying the thick coelomyarian musculature (black arrow), and the intestine (blue arrow). A uterine tube (green arrow) containing eggs (arrowhead) is also visible. H&E staining. c Higher magnification of the transverse section of an adult female parasite (P) lined by neutrophils (stars), necrotic debris, mononuclear inflammatory cells, multinucleated giant cells (arrows), and collagen fibers. H&E staining. d Transverse section of a calcified dead adult nematode (CP) surrounded by inflammatory cells that underwent coagulative necrosis, and connective tissue. H&E staining. Scale-bars: a–d, 100 µm. Abbreviations: IR, inflammatory reaction; P, parasite; CP, calcified parasite. [file 13071_2020_4123_MOESM4_ESM.jpg]
